# Tobacco Use, Stigma, and Coping in Lung Cancer: A Systematic Review of Their Psychosocial Interactions and Clinical Implications

**DOI:** 10.3390/curroncol33070408

**Published:** 2026-07-09

**Authors:** Anais Sánchez-Ros, Francisco Tomás-Aguirre, Marcelino Pérez-Bermejo, María Teresa Murillo-Llorente, María Ester Legidos-García, Ignacio Ventura, Teresa Mayordomo-Rodriguez

**Affiliations:** 1 Doctoral School, Catholic University of Valencia San Vicente Mártir, c/Quevedo, 2, 46001 Valencia, Spain; anais.sanchez@mail.ucv.es; 2 SONEV Research Group, Faculty of Medicine and Health Sciences, Catholic University of Valencia San Vicente Mártir, C/Quevedo No. 2, 46001 Valencia, Spain; paco.tomas@ucv.es (F.T.-A.); mt.murillo@ucv.es (M.T.M.-L.); ester.legidos@ucv.es (M.E.L.-G.); 3 Faculty of Medicine and Health Sciences, Catholic University of Valencia San Vicente Mártir, C/Quevedo No. 2, 46001 Valencia, Spain; ignacio.ventura@ucv.es; 4 Departamento Psicología de la Personalidad, Tratamientos y Metodología, Avda de la Ilustración, 2, 46010 Valencia, Spain; teresa.mayordomo@ucv.es

**Keywords:** lung cancer, stigma, coping, psychological distress, quality of life, social support, systematic review

## Abstract

Background: Lung cancer carries a high psychosocial burden. Tobacco use, the stigma attached to the disease, and coping strategies are thought to interact and shape psychological outcomes, yet they have rarely been examined together. This review aimed to synthesise the evidence on the relationship between tobacco use, lung cancer stigma, and coping, and how these factors interact and influence patients’ psychological outcomes. Methods: Following the PRISMA 2020 guideline, PubMed/MEDLINE and Dialnet were searched (window 2014–April 2026) for empirical studies conducted in adults with lung cancer that addressed stigma, coping, or relevant psychological outcomes (e.g., anxiety, depression, distress, or quality of life). Study selection and data extraction were performed independently by two reviewers, with discrepancies resolved by consensus and, where needed, by a third reviewer. Methodological quality was appraised with design-specific tools (JBI for cross-sectional and cohort studies, CASP for qualitative studies, and COSMIN-oriented criteria for the psychometric study). Given the clinical and methodological heterogeneity, a structured narrative synthesis was conducted following the SWiM guideline. The protocol was registered in the Open Science Framework. Results: Twenty-four studies were included. Stigma was prevalent and consistently associated with depression, anxiety, distress, and poorer quality of life, with longitudinal evidence indicating that stigma precedes and predicts distress. Internalised stigma (guilt, shame, self-blame) was the facet most strongly linked to depression and anxiety. Smoking history graded stigma intensity (current > former > never smokers) but did not determine it, since clinically significant stigma also affected never-smokers. Adaptive coping (e.g., fighting spirit, positive reappraisal) and social support were consistently associated with better psychological adjustment and quality of life, while maladaptive coping (e.g., helplessness, avoidance, anxious preoccupation) was associated with worse outcomes; cross-sectional evidence further indicated that coping modes mediated the relationship between stigma and quality of life and that social support and self-compassion attenuated the impact of stigma on distress. Conclusions: Internalised stigma is a central, modifiable psychosocial stressor in lung cancer that affects smokers and never-smokers alike. Systematic screening for stigma, coping, and social support, together with non-stigmatising care, is warranted.

## Simple Summary

Lung cancer is one of the most common and deadly cancers, and those who develop it often carry a heavy emotional burden. Many feel blamed for their illness because it is so closely linked in the public mind to smoking, and this sense of being judged, known as stigma, can deepen their distress. In this systematic review we brought together the published research on how smoking history, the stigma attached to the disease, and the ways patients cope are connected, and how together they shape emotional well-being. We found that stigma, especially when patients turn it against themselves through guilt and shame, is strongly tied to anxiety, depression, and poorer quality of life, and affects even patients who never smoked. We also found that good coping skills and social support protect well-being. These findings can help clinicians address the emotional needs of people with lung cancer.

## 1. Introduction

According to the GLOBOCAN 2022 estimates, produced by the International Agency for Research on Cancer (IARC), lung cancer had the highest cancer mortality rate, with approximately 1,817,469 deaths, representing around 18.7% of all cancer deaths [1]. In this context, lung cancer remains one of the greatest challenges in global public health, not only because of its high mortality but also because of a clinical and psychosocial complexity that distinguishes it from other cancers [2].

The first symptoms of lung cancer are usually nonspecific and appear in advanced stages, which hinders its diagnosis [3]. As a result, the diagnosis is often made late and the prognosis is more severe [3,4]. Despite advances in detection and treatment, it remains one of the cancers with the lowest survival rate [5]. This diagnostic delay is not due solely to clinical factors but also to psychosocial processes such as the minimisation of symptoms, fear of the diagnosis, or avoidance of the healthcare system, especially in people with a smoking history [6].

According to the World Health Organization, between 30% and 50% of cancer cases could be prevented through healthy lifestyles [7]. In lung cancer, the main risk factor is tobacco use: it is estimated that between 80% and 90% of deaths from this cancer are related to smoking [8]. In fact, the risk of developing lung cancer in smokers is between 10 and 20 times higher than in non-smokers, in proportion to the intensity and duration of consumption [9]. Beyond its aetiological role, tobacco use has been linked to psychological processes related to emotion regulation and stress coping; the evidence indicates that nicotine dependence is associated with difficulties in regulating emotions and with greater reliance on maladaptive coping strategies [10,11,12].

Cancer is, moreover, a stigmatised disease, largely because of its association with suffering and with a higher probability of death than other diseases [13]. This is compounded by the visible physical changes that frequently accompany the disease and its treatments, such as hair loss, scarring, or deterioration of physical appearance [14]. Cancer-related stigma can be conceptualised as a multidimensional phenomenon comprising perceived, experienced, and internalised components, which significantly affects the patient’s psychological and social well-being [15].

Lung cancer is one of the most studied cancers from the perspective of stigma, as it is perceived as a disease whose cause depends largely on the individual’s own behaviour, which is linked to the widespread belief that all lung cancers are due to smoking and to the consequent blaming of patients [16,17]. This blame is reinforced by an additional stigma directed at tobacco use itself, which is often framed as a voluntary choice [18] and ignores the addictive nature of nicotine and its high dependence potential [19]. Various studies have shown that this stigma is associated with higher levels of depression and anxiety and worse quality of life, as well as with delays in seeking medical care and lower adherence to cancer treatment [20,21,22,23]. Moreover, it can take internalised forms that increase feelings of guilt and shame and hinder psychological adjustment [17,23].

Cancer patients, especially those living with the disease in advanced stages, experience numerous physical and psychological symptoms that impair their quality of life [24]. On the psychological level, it is estimated that between 20% and 50% of cancer patients present clinically significant levels of emotional distress [25]. The National Comprehensive Cancer Network (NCCN), a US-based alliance of leading cancer centres, has defined this distress as a maladaptive emotional state that interferes with the ability to cope with the disease.

Distress is a frequent reaction in cancer patients, but it tends to manifest more intensely in lung cancer [26]. In fact, this cancer has one of the highest prevalences of depression among the various types of cancer: approximately 13% of patients meet criteria for major depression assessed by clinical interview (the highest figure among the major cancers) whereas clinically significant depressive symptoms, assessed by self-report, affect about one in three patients; in addition, a substantial proportion of these cases remain undiagnosed and untreated [27]. Depression, in turn, has been associated with a worse prognosis in these patients [28]. In this context, lung cancer stigma has been identified as a factor that contributes to increasing distress, acting as an additional psychosocial stressor that amplifies the emotional impact of the disease [23,26].

Anxiety is likewise a relevant component of the psychological impact of cancer, closely linked to the emotional response to the diagnosis and to the patient’s decision-making capacity [29]. Although a certain degree of anxiety may be adaptive (by mobilising resources to cope with the disease) an excessive level may lead to significant distress that interferes with clinical decisions, hinders treatment adherence, and reduces quality of life [20,30].

In this scenario, coping takes on a fundamental role. In the oncological setting it is understood as the set of cognitive and behavioural responses that enable the patient to deal with the disease, modulated by the meaning it has for them and by their perception of control [31,32]. An appropriate coping strategy can markedly improve emotional adjustment and quality of life [32].

The most widely accepted theoretical framework is that of Lazarus and Folkman, which defines coping as the constantly changing cognitive and behavioural efforts aimed at managing demands perceived as exceeding the individual’s resources. In this model, strategies are grouped into two broad categories: problem-focused, which seek to act on the source of stress, and emotion-focused, aimed at regulating the emotional response [33,34].

In lung cancer, where the psychological impact is especially intense, models such as Moorey and Greer’s survival schema help us to understand the different ways of coping with the disease. Based on the meaning attributed to the diagnosis, the predominant emotions, and the sense of control, five main coping styles are described (fighting spirit, avoidance-denial, fatalism, helplessness, and anxious preoccupation) which are usually grouped into active or passive responses [32,35].

Active strategies, such as fighting spirit, are associated with better psychological adjustment and greater well-being, whereas passive ones, such as helplessness or anxious preoccupation, tend to be related to worse emotional outcomes [32]. In lung cancer patients, maladaptive coping strategies have been associated with higher levels of distress and worse quality of life, which underscores the clinical relevance of this construct [29,36].

Taken together, the evidence suggests a complex relationship between tobacco use, lung cancer stigma, and the patient’s psychological responses. A smoking history may increase the perception of guilt and stigmatisation, which in turn would contribute to greater emotional distress and condition the coping strategies used. However, the specific mechanisms that explain this interaction have not been sufficiently clarified.

Although there are numerous studies on coping in cancer patients, reviews focused specifically on the interaction between stigma, tobacco use, and coping in lung cancer are scarce. Earlier reviews [17] have mapped lung cancer stigma in isolation or focused on its association with single outcomes such as quality of life or treatment delay, but they predate the development and validation of the Lung Cancer Stigma Inventory (LCSI) [21,23], the publication of longitudinal evidence indicating that stigma precedes and predicts distress [22,37,38], the formal articulation of self-compassion as a moderator of the stigma–distress pathway [22], and the recent expansion of Asian and Latin American evidence, together with earlier qualitative evidence from China [39,40,41,42,43,44,45]. Together, these developments justify a new, updated synthesis that integrates the three constructs in a single conceptual framework and incorporates the most recent cross-cultural evidence. Moreover, the literature tends to address these factors in isolation, without integrating their joint impact on psychological outcomes. This gap is especially relevant from a clinical perspective, as it limits the development of specific and effective psycho-oncological interventions.

Therefore, the present work aims to systematically synthesise the available evidence on the relationship between tobacco use, lung cancer stigma, and coping strategies, and to analyse how these factors interact and influence patients’ psychological outcomes. A systematic review approach was chosen, in preference to a scoping review, in accordance with the criteria proposed by Munn et al. [46]. Whereas scoping reviews are indicated when the purpose is to map a broad body of literature, clarify concepts, or identify research gaps, systematic reviews are appropriate when the aim is to synthesise and critically appraise the evidence in order to answer a clinically meaningful question. Our review fits the second profile: it addresses a focused question with explicit a priori eligibility criteria, includes formal risk-of-bias appraisal, and seeks to inform clinical decision-making on the assessment and management of psychosocial distress in lung cancer. Reporting followed the PRISMA 2020 statement, and the protocol was registered prospectively in the Open Science Framework.

## 2. Materials and Methods

### 2.1. Study Design, Protocol, and Registration

A systematic literature review was conducted in accordance with the recommendations of the PRISMA 2020 statement (Preferred Reporting Items for Systematic Reviews and Meta-Analyses) [47], whose 27-item checklist was applied throughout all phases of the process. The aim was to identify, critically appraise, and synthesise the available scientific evidence on the relationship between tobacco use, lung cancer stigma, and coping strategies, as well as their impact on patients’ psychological burden.

The review protocol was registered on the Open Science Framework (OSF; https://osf.io; accessed on 2 June 2026), under doi 10.17605/OSF.IO/NJG68. The full protocol, the search equations for each database, the eligibility criteria, the data extraction form, and the completed PRISMA 2020 checklist are deposited in that registry, in order to ensure the transparency and reproducibility of the study.

Given the methodological heterogeneity of the included studies (which comprise observational, qualitative, and psychometric designs) and the diversity of variables and measurement instruments used, a meta-analysis was not considered appropriate. Instead, a structured narrative synthesis was carried out, whose elaboration and presentation followed the recommendations of the SWiM guideline (Synthesis Without Meta-analysis) [48], in order to ensure traceability between the extracted data, the synthesis performed, and the conclusions.

### 2.2. Research Question and Conceptual Framework

The review was designed to answer the following question: what evidence exists on the relationship between tobacco use, lung cancer stigma, and coping strategies, and how do these factors interact in patients’ psychological burden?

Given the mixed nature of the evidence (quantitative and qualitative studies), the question was structured using the SPIDER framework (Sample, Phenomenon of Interest, Design, Evaluation, Research type), which is more suitable than the PICO model for reviews that integrate qualitative research, although the latter was used in a complementary way to guide the search for quantitative studies [49]. The components were defined as follows:

- Sample: adults (≥18 years) with a diagnosis of lung cancer.
- Phenomenon of Interest: tobacco use or smoking history, stigma (perceived, internalised, anticipated, and disclosure-related), guilt, shame, and coping strategies.
- Design: observational (cross-sectional and longitudinal), qualitative, mixed, and psychometric studies.
- Evaluation: distress, anxiety, depression, quality of life, emotion regulation, risk perception, psychological adjustment, and help-seeking behaviour.
- Research type: quantitative, qualitative, and mixed.

### 2.3. Information Sources and Search Strategy

The literature search was carried out between April and May 2026 in the PubMed/MEDLINE and Dialnet databases. PubMed/MEDLINE was selected as the principal biomedical source because it indexes many of the journals in which primary studies on lung cancer stigma, coping, and psychological adjustment are published, including psycho-oncology, behavioural medicine, oncology, and cancer nursing journals. Dialnet was added to capture peer-reviewed Spanish-language literature, which is under-represented in major international databases and is relevant for the cultural transferability of the findings to Spanish-speaking clinical contexts. This database strategy was complemented by backward and forward citation searching in order to improve retrieval of psychosocial studies located at the interface between oncology and behavioural sciences. Nevertheless, the absence of psychology-specific and multidisciplinary databases such as PsycINFO, CINAHL, Scopus, or Web of Science is acknowledged as a limitation of the review. The elaboration and reporting of the strategy followed the PRISMA-S extension for the reproducible documentation of searches [50]. Institutional sources (WHO [7], IARC [1], and AECC [51]) were also consulted solely for epidemiological contextualisation, without incorporating them into the synthesis of empirical evidence.

The strategy combined controlled terms (MeSH descriptors in PubMed/MEDLINE) and free-text terms relating to the axes of the study (lung cancer, tobacco use, stigma, and coping) linked by the Boolean operators AND and OR and adapted to the syntax of each database. The main combination in PubMed/MEDLINE was: (lung cancer) AND (stigma OR coping OR psychological outcomes-distress, depression, anxiety, or quality of life). In database syntax: (“Lung Neoplasms”[Mesh] OR “lung cancer”[tiab] OR “lung carcinoma”[tiab] OR NSCLC[tiab] OR SCLC[tiab]) AND (“Social Stigma”[Mesh] OR stigma*[tiab] OR shame[tiab] OR guilt[tiab] OR “Adaptation, Psychological”[Mesh] OR coping[tiab] OR “Stress, Psychological”[Mesh] OR distress[tiab] OR depress*[tiab] OR anxiety[tiab] OR “Quality of Life”[Mesh] OR “quality of life”[tiab]). The tobacco block (“Smoking”[Mesh] OR smoking[tiab] OR tobacco[tiab] OR smoker*[tiab]) was used as an optional term to refine the third axis, not as a mandatory filter, so as not to exclude studies on stigma or coping or patients who have never smoked. The complete equations for each database are provided in the Supplementary Material (Supplementary Materials File S1) and in the OSF registry; the completed PRISMA 2020 checklist is provided as Supplementary Materials File S2.

### 2.4. Eligibility Criteria

The inclusion criteria were: (a) original studies with empirical data; (b) published between 2014 and April 2026 (the start date was chosen to coincide with the consolidation of validated lung-cancer-specific stigma measurement, in the period immediately preceding and following the development of the Lung Cancer Stigma Inventory [21], which redefined the empirical study of internalised stigma in this population); (c) written in English or Spanish; (d) conducted in an adult population; and (e) addressing at least one of the axes of the study (tobacco use or smoking history, lung cancer stigma, or coping strategies) or relevant psychological variables (anxiety, depression, distress, or quality of life) in relation to lung cancer. The decision to include studies that addressed at least one (and not necessarily all three) of the axes was deliberate: requiring the simultaneous presence of stigma, coping, and smoking history would have yielded a very small and biased subset of studies (essentially restricted to a single research group), and would have prevented the integrative synthesis that the review aims to achieve. Including single-axis studies allowed us to triangulate the evidence across the three constructs and to identify their points of convergence and divergence in a structured narrative synthesis. Observational, qualitative, and mixed designs were accepted.

The exclusion criteria were: (a) studies focused exclusively on family members or caregivers; (b) studies on screening programmes, cancer survivors, or therapeutic interventions without psychosocial variables; (c) purely biomedical studies; and (d) reviews, editorials, letters to the editor, conference communications, and documents without empirical data.

### 2.5. Study Selection Procedure

All identified records were imported into a reference manager for the automatic removal of duplicates, followed by manual screening to detect duplicates not identified by the software and erroneous records. Selection proceeded in two phases. In the first, two reviewers independently examined titles and abstracts to determine the potential relevance of each reference. In the second, the full text of potentially eligible articles was obtained and their compliance with the inclusion and exclusion criteria was assessed. Agreement between reviewers was evaluated and discrepancies were resolved by consensus; when this was not possible, a third reviewer intervened. The selection process, with the number of records identified, screened, assessed in full text, excluded (with reasons), and included, is represented in the PRISMA 2020 flow diagram [47].

### 2.6. Data Extraction

Data extraction was performed using a standardised, previously piloted form, independently and in duplicate by two reviewers (A.S.-R. and F.T.-A.), with discrepancies resolved by consensus and, when this was not possible, by a third reviewer (T.M.-R.). For each study the following were recorded: authorship and year, country, design, sampling frame and sample size, sociodemographic and clinical characteristics of the sample, smoking history, psychological variables analysed, measurement instruments and their psychometric properties, main results, and limitations noted by the authors. In cases of insufficient or ambiguous information, the protocol envisaged requesting additional data from the corresponding authors; in practice, no such request was deemed necessary, as the published reports of all 24 included studies contained sufficient information for the variables of interest. Studies excluded at the full-text stage on account of insufficient information were excluded because their reports lacked the design or outcome data required by the eligibility criteria, not because individual values were missing.

### 2.7. Variables of Interest

The primary variables were lung cancer stigma, analysed in its perceived, internalised, anticipated, and disclosure-related dimensions, as well as in terms of guilt and shame; coping strategies, classified as adaptive (e.g., fighting spirit or positive reappraisal) and maladaptive (e.g., helplessness, fatalism, avoidance, or anxious preoccupation); and smoking history, classified as never smoker, former smoker, or current smoker.

The secondary variables included distress, anxiety, depression, quality of life, emotion regulation, rumination, risk perception, and help-seeking behaviour.

### 2.8. Assessment of Methodological Quality and Risk of Bias

Methodological quality and risk of bias were assessed using design-specific tools. For observational studies, the Joanna Briggs Institute (JBI) critical appraisal checklists were used: the one for analytical cross-sectional studies and the one for cohort studies, the latter for longitudinal designs [52]. Qualitative studies were appraised with the CASP Qualitative Checklist [53] and the psychometric study with orientative criteria of the COSMIN initiative for measurement instruments. The use of the Mixed Methods Appraisal Tool (MMAT), version 2018, was envisaged for mixed-methods studies [54], although ultimately none meeting the inclusion criteria was identified. The MMAT was nevertheless declared in the protocol because it was considered probable a priori that mixed-methods studies would be retrieved given the heterogeneous design of psycho-oncology research; reporting all the appraisal tools planned in advance, including those ultimately not used, is consistent with PRISMA 2020 recommendations on the transparency of methodological choices. The assessment was performed independently and in duplicate by two reviewers (A.S.-R. and F.T.-A.); inter-rater agreement at the item level was high (overall pooled agreement > 90%), with discrepancies resolved by consensus and, where this was not possible, by a third reviewer (T.M.-R.). No study was excluded on the basis of its quality; instead, methodological limitations were incorporated into the interpretation of the results, weighting more strongly those findings that derived from studies of higher methodological quality and from longitudinal designs. The main sources of bias identified were the use of self-reports, the predominance of cross-sectional designs, and the limited representativeness of some samples.

### 2.9. Data Synthesis

Given the clinical and methodological heterogeneity of the included studies (which combine observational (cross-sectional and longitudinal), qualitative, and psychometric designs, with diverse variables and measurement instruments) a meta-analysis was not considered appropriate. Instead, a structured narrative synthesis was carried out, elaborated and reported in accordance with the recommendations of the SWiM (Synthesis Without Meta-analysis) guideline [48].

The findings were organised around the three central constructs that define the research question (stigma, coping strategies, and smoking history) operationalised in the following three thematic axes: (1) lung cancer stigma and its psychological impact (depression, anxiety, distress, and quality of life); (2) coping strategies and their relationship with adjustment and quality of life; and (3) smoking history, stigma, and help-seeking behaviour. Evidence from quantitative and qualitative studies was integrated within each axis through a convergent synthesis approach. The convergent approach was operationalised in four steps. First, results from quantitative studies (statistical associations, effect sizes when reported, and direction of relationships) and results from qualitative studies (descriptive themes and subthemes) were extracted in parallel for each axis. Second, quantitative findings were tabulated and characterised in terms of direction, magnitude (when available), and consistency across studies; qualitative findings were coded into themes that could be linked to the same conceptual axis (e.g., narratives of self-blame as qualitative evidence of internalised stigma). Third, themes and statistical associations were juxtaposed and compared within each axis to identify points of convergence (qualitative themes corroborating quantitative associations, such as “guilt and shame” in qualitative narratives mirroring the depression–internalized stigma association quantified by [38,55]), points of complementarity (qualitative findings adding mechanistic detail to quantitative associations), and points of divergence (qualitative descriptions inconsistent with the predominant quantitative pattern). Fourth, the integrated findings were narratively synthesised, weighting longitudinal and higher-quality designs more heavily and using qualitative evidence to refine the interpretation rather than to override quantitative effect direction. This integrative procedure follows the Joanna Briggs Institute guidance for convergent integrated mixed-methods synthesis and the SWiM recommendations on reporting narrative syntheses without meta-analysis.

For each axis, the results were tabulated (authorship, design, population, variables, instruments, and main findings) and synthesised in a structured manner, describing the direction, the magnitude (when available) and the consistency of the associations across studies, without using simple vote counting as the sole synthesis criterion. In the interpretation, the design of the studies (giving greater weight to longitudinal evidence), the risk of bias, and the consistency of the results were weighted. From this organisation, an integrative interpretation was developed that articulates the relationship between stigma, coping, and smoking history, presented as a provisional explanatory model of psychological adjustment to lung cancer, subject to the limitations of the available evidence.

### 2.10. Assessment of the Certainty of the Evidence

Confidence in the synthesised findings was assessed in a structured manner following the principles of the JBI Manual for Evidence Synthesis [52], taking into account the design and risk of bias of the studies, the coherence and consistency of the results, and the precision and relevance of the available evidence for each of the three thematic axes.

## 3. Results

### 3.1. Selection and Characteristics of the Included Studies

**Figure 1:**
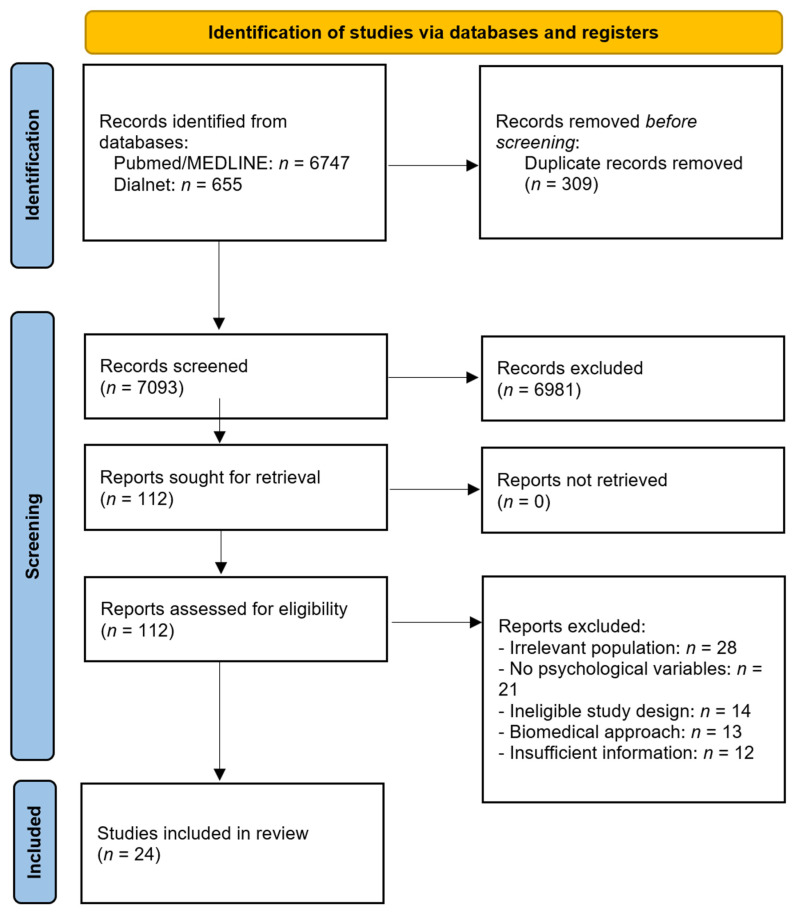
PRISMA 2020 flow diagram.

After the search, screening, and verification process, 24 studies were included in the qualitative synthesis, all of them conducted in lung cancer populations (Table 1). The identification, screening, and selection process is represented in the PRISMA 2020 flow diagram (Figure 1). The results of the methodological quality assessment are presented in Table 2, Table 3 and Table 4.

#### Psychometric Study (Appraisal with COSMIN-Oriented Criteria)

Hamann et al. (2018) [21] (Development and preliminary validation of the Lung Cancer Stigma Inventory, LCSI). As this is an instrument-development study, appraisal with the above tools is not applicable; it is appraised with orientative COSMIN criteria. The available evidence indicates a multiphase development with patient input (content validity), evaluation of the instrument’s structure and of internal consistency, with adequate psychometric properties. Overall assessment: adequate psychometric quality for an instrument-development study.

### 3.2. Lung Cancer Stigma and Psychological Impact

The included studies consistently indicate that lung cancer patients present high levels of stigma, conceptualised as a multidimensional phenomenon with perceived and internalised components [21,55]. Stigma is associated with a greater presence of depression [23,57], anxiety [55], psychological distress [40,57], and worse quality of life [40,41,57]. The longitudinal studies provide directional evidence: baseline stigma predicts increases in distress at 3 and 6 months [37] and worse psychological adjustment over time [22,38]. The internalised component (guilt, shame, and self-blame) is the facet most consistently linked to depression and anxiety [38,55]. Evidence from diverse cultural contexts confirms and extends this pattern: stigma worsens quality of life through distress [40] and through coping modes [41], and, from a qualitative perspective, Chinese patients identify various sources of stigma (tobacco, loss of working capacity, or deterioration of self-image) and resort to strategies such as concealing the illness [42]. Likewise, patient–provider communication is related to perceived stigma [58], whereas self-compassion attenuates the relationship between stigma and distress [22]. In advanced disease, the burden of anxiety, depression, and distress is particularly high and is associated with worse quality of life [39].

In terms of prevalence, the cross-sectional evidence converged on substantial figures: Chambers et al. [57] reported clinically significant anxiety in 49%, depression in 41%, and distress in 51% of their sample (*n* = 151), figures broadly consistent with the proportions documented by Morrison et al. [26] (*n* = 2205 newly diagnosed patients), by González-Ling et al. [39] in Latin American advanced-disease populations (*n* = 204), and by Lim et al. [40] in Korean patients (*n* = 184). Internalised stigma scores measured with the Lung Cancer Stigma Inventory (LCSI) reached or exceeded the clinical threshold of 38 in approximately one quarter to one third of patients across the validation samples [16,21,23], with a substantial proportion of never-smokers (around 60% in the largest sample [16]) also reaching that threshold. Across studies that reported them, correlations between stigma and depression typically ranged in the moderate band (Pearson’s r approximately 0.30–0.50), and similar magnitudes were observed for the stigma–distress and stigma–quality-of-life associations [21,23,40,55,57]. Although the diversity of instruments precluded pooled estimates, the direction and approximate magnitude of these associations were highly consistent across countries (United States, Australia, Poland, Netherlands, Mexico, China, South Korea, and Taiwan), supporting the robustness of the stigma–distress relationship as a cross-cultural phenomenon. Longitudinal studies further constrained the direction of effect: in Rose et al. [37], baseline stigma significantly predicted distress trajectories at 3 and 6 months after diagnosis, and in Williamson et al. [22,38] stigma facets predicted poorer psychological adjustment at 12 weeks, with self-compassion emerging as a statistically significant moderator of these effects. Taken together, these findings indicate that stigma is not only highly prevalent in lung cancer but also consistently associated with adverse psychological outcomes of clinically relevant magnitude, with longitudinal evidence supporting a temporal precedence of stigma over distress.

### 3.3. Coping Strategies, Social Support, and Quality of Life

The included studies show that coping strategies influence emotional state and quality of life. The type of coping is related to levels of anxiety and depression [29], and maladaptive (especially avoidant) styles are associated with worse adjustment and lower quality of life [36], while the integration of psychological factors into profiles makes it possible to identify the most vulnerable patients [61]. Recent evidence refines the role of coping by situating it as a mediating mechanism: coping modes mediate the relationship between stigma and quality of life [41] and between illness perception and distress [44]. The qualitative contribution enriches this picture by describing a broad repertoire of responses (social support, reorientation towards the positive, avoidance, and spiritual resources) in women with incurable disease [60]. These results are consistent with the classic theoretical frameworks of coping [33,34] and with the schema of response styles to cancer [35], and reinforce the idea that coping is a process amenable to intervention.

When the extracted data are mapped onto the five coping styles described by Moorey and Greer [35] (fighting spirit, avoidance-denial, fatalism, helplessness, and anxious preoccupation), the findings of the included studies show a coherent pattern that can be summarised as follows. (1) Fighting spirit, captured in the included studies primarily through the mini-MAC constructive subscale [29,36] and through qualitative themes of active engagement and positive reframing [60], was consistently associated with lower anxiety and depression and better quality of life; in Jankowska-Polańska et al. [29], constructive coping styles (which include fighting spirit) showed protective associations with both anxiety and depression scores (HADS) in NSCLC patients. (2) Anxious preoccupation, identifiable in the mini-MAC destructive subscale and in qualitative descriptions of intrusive worry, was associated with higher distress and lower quality of life in cross-sectional analyses [29,36,44,61]. (3) Helplessness/hopelessness, also captured in the mini-MAC destructive subscale and qualitatively expressed as the perception of having lost control over the disease, was the destructive style most strongly associated with worse psychological outcomes [29,36], and contributed to the profiles of patients with the poorest quality of life identified by van Montfort et al. [61]. (4) Fatalism produced mixed associations across studies: it was sometimes grouped with constructive responses and sometimes with destructive ones depending on the cultural meaning attributed to it (e.g., resignation versus acceptance). In studies conducted in Asian contexts [41,43,44,60], constructs conceptually close to fatalism were sometimes embedded within broader frameworks of acceptance, spirituality, or meaning-making; however, because fatalism was not measured uniformly across studies, this interpretation should be approached with caution. (5) Avoidance-denial, including concealment of the illness, was associated with worse psychological adjustment and quality of life [36,38,42], and Liu et al. [42] described concealment of the illness as a frequent strategy used by Chinese patients to cope with anticipated and enacted stigma. Across the body of evidence, the contrast between fighting spirit and positive reappraisal on the one hand, and helplessness, anxious preoccupation, and avoidance on the other, was the most discriminating dimension for psychological outcomes, in line with the Lazarus and Folkman [33,34] distinction between problem-focused and emotion-focused strategies and with the broader cancer-specific schema of Moorey and Greer [35].

### 3.4. Smoking History, Stigma, and Help-Seeking

The evidence on the relationship between smoking history and stigma is mixed but, taken as a whole, suggests a graded rather than a categorical association, with two related observations. First, in the studies that did detect differences by smoking status [16], stigma scores (total, internalised, and perceived) were higher in patients who smoked at the time of diagnosis than in former smokers, and in former smokers higher than in those who had never smoked. Second, and importantly, clinically significant stigma was also documented in a high proportion of patients who had never smoked (around 60% in the largest sample [16]) and was reported by these patients in qualitative interviews [62], which qualifies the idea that only those with a history of tobacco consumption experience internalised stigma. Several studies, however, did not find statistically significant differences across smoking-status categories, so the graded pattern should be interpreted as a tendency rather than a universal finding. Two qualitative studies provided complementary insights into how patients themselves discuss smoking in research interviews: Hajdarevic et al. [59] described how patients managed disclosure and avoidance of the topic of smoking in their accounts to the interviewers, illustrating how stigma shapes the narrative that patients construct about the illness; Black et al. [62] showed that patients with and without a smoking history differed in how they appraised their first respiratory symptoms and decided whether to seek medical care. Greater stigma was also associated with longer delays in seeking medical care [56]. These findings, taken together, indicate that smoking history grades the intensity of stigma without being its necessary condition, and that it modulates pre-diagnostic behaviour at the symptom-appraisal and help-seeking stages.

## 4. Discussion

This systematic review, which integrates 24 studies conducted in lung cancer populations, confirms that stigma, coping, and smoking history configure a complex psychosocial scenario, and allows a substantial nuancing of the way these factors relate to each other. Four central ideas emerge from the synthesis: stigma is a prevalent phenomenon that is robustly associated with distress, depression, anxiety, and worse quality of life; its internalised component (guilt and shame) is the most harmful; stigma does not depend exclusively on smoking history; and both coping and, notably in the most recent evidence, social support modulate the patient’s adjustment. The incorporation of Asian and Latin American studies reinforces these findings across diverse cultural contexts.

### 4.1. Stigma as a Central Psychosocial Factor

The included studies agree that lung cancer patients experience high levels of stigma, conceptualised as a multidimensional construct measurable with validated instruments such as the Lung Cancer Stigma Inventory [21]. This stigma is consistently associated with depression [23,57], anxiety [55], distress [40,57], and a deterioration in quality of life [40,41,57]. Beyond cross-sectional associations, the longitudinal studies provide a relevant degree of directionality: baseline stigma predicts subsequent increases in distress [37] and worse psychological adjustment over time [22,38], which attenuates (though does not eliminate) the limitation of cross-sectionality. Evidence from diverse cultural contexts reproduces this pattern: stigma worsens quality of life through distress [40] and through coping modes [41], and qualitative studies in non-Anglo-Saxon contexts, such as the Chinese one, describe the sources and experiences of stigma (linked to tobacco, loss of working capacity, and deterioration of self-image) and coping strategies such as concealing the illness [42], which adds a sociocultural dimension that has been little explored to date.

Among the various facets of stigma, the internalised one (self-blame, guilt, and shame) emerges as the most closely linked to depressive and anxious symptomatology [38,55]. This finding shifts the focus of intervention towards the patient’s self-appraisal processes. Consistent with this, self-compassion attenuates the relationship between stigma and distress [22], and the quality of patient–provider communication is associated with the intensity of perceived stigma [58]. In advanced and metastatic disease, the burden of anxiety, depression, and distress is particularly high [39], which reinforces the need for systematic psychosocial screening.

### 4.2. Smoking History: A Graded, Not Categorical, Relationship

The relationship between smoking history and stigma proved to be more nuanced than is usually assumed. On the one hand, stigma is not exclusive to patients with a history of consumption: it is frequently documented, at clinically significant levels, also in those who have never smoked (around 60% in one of the studies with the largest sample size) [16]. On the other hand, several studies have found that, where differences across smoking-status categories are observed, stigma scores tend to be higher in current smokers than in former smokers and higher in former smokers than in never-smokers [16]. The evidence is, however, mixed: a subset of studies did not detect statistically significant differences by smoking status, and the pattern should therefore be interpreted as a tendency rather than as a universal categorical effect. Taken together, the evidence supports the view that smoking history grades the intensity of stigma in some samples and contexts without being its necessary condition; once present, clinically significant stigma affects the entire lung cancer population, including those who have never smoked.

Smoking history also plays a differentiated role in pre-diagnostic behaviour. Qualitative evidence shows that patients with and without a history of consumption interpret and respond differently to the first symptoms [62], and that stigma conditions the way smoking is addressed (or avoided) in the patient’s accounts during qualitative research interviews [59]. In turn, greater stigma has been associated with a delay in seeking medical care [56]. Taken together, the evidence suggests that smoking may influence both the intensity of stigma and, more consistently, symptom appraisal and help-seeking behaviour, although the magnitude of these influences varies across studies and contexts.

### 4.3. Coping and Social Support as Modulators of Adjustment

The synthesis confirms that coping strategies modulate emotional state and quality of life. The type of coping is related to levels of anxiety and depression [29], and maladaptive (especially avoidant) styles are associated with worse adjustment and lower quality of life [36], while the integration of psychological factors into profiles makes it possible to identify the most vulnerable patients [61]. Recent evidence refines the role of coping by situating it as a mediating mechanism: coping modes mediate the relationship between stigma and quality of life [41] and between illness perception and distress [44]. The qualitative contribution enriches this picture by describing a broad repertoire of responses (social support, reorientation towards the positive, avoidance, and spiritual resources) in women with incurable disease [60]. These results are consistent with the classic theoretical frameworks of coping [33,34] and with the schema of response styles to cancer [35], and reinforce the idea that coping is a process amenable to intervention.

A finding that emerges strongly from the most recent studies is the role of social support as a protective and modulating factor. Social support buffers the effect of depression on quality of life in patients undergoing chemotherapy [45], moderates the relationship between stigma and quality of life (such that support from friends attenuates its impact) [40] and, together with coping style, conditions the patient’s psychological state [43]. Coping and social support thus appear as two modifiable and complementary targets, suggesting that the most effective interventions will be those that act simultaneously on the patient’s internal (coping, self-compassion) and external (social support) resources.

### 4.4. Towards a Revised Integrative Model

**Figure 2:**
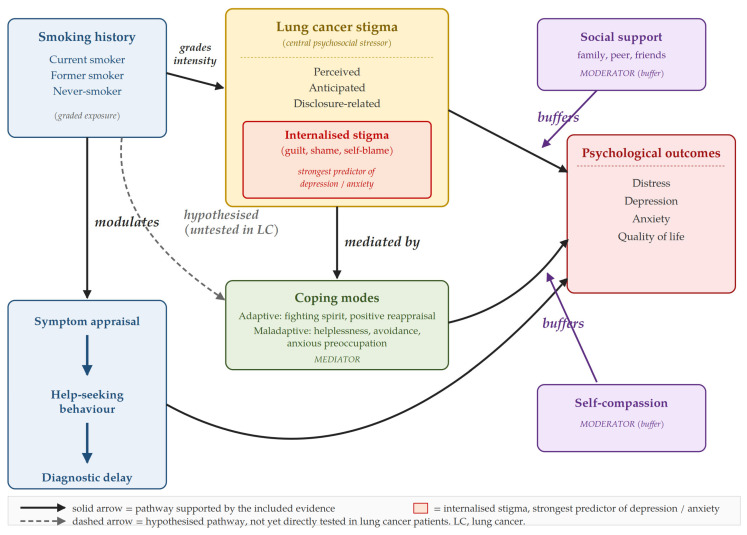
Provisional integrative model of the relationship between smoking history, lung cancer stigma, coping, social support, self-compassion, and psychological outcomes (distress, depression, anxiety, quality of life), based on the synthesised evidence. Lung cancer stigma is positioned as the central psychosocial stressor; its internalised facet (guilt, shame, self-blame) is highlighted as the pathway most strongly linked to depression and anxiety. Smoking history grades the intensity of stigma and modulates symptom appraisal, help-seeking behaviour, and diagnostic delay. Coping modes mediate the relationship between stigma and psychological outcomes, while social support and self-compassion act as buffers on that pathway. The dashed arrow from smoking history to coping modes represents the hypothesised emotion-regulation pathway, not yet directly tested in lung cancer patients. Box fill colours indicate the functional role of each construct: gold denotes the central psychosocial stressor (lung cancer stigma); blue denotes smoking history and its associated exposure pathway (smoking status, perceived/anticipated/disclosure-related stigma, symptom appraisal, help-seeking behaviour, and diagnostic delay); orange denotes internalised stigma, the facet most strongly linked to depression and anxiety; green denotes coping modes (mediator); purple denotes social support and self-compassion (moderators/buffers); and red denotes the psychological outcomes (distress, depression, anxiety, quality of life). Text colour within each box matches its fill colour, and the red callout text on the arrow from internalised stigma to the outcomes highlights this as the pathway most strongly supported by the included evidence. Solid arrows = pathway supported by the included evidence; dashed arrow = hypothesised pathway. LC, lung cancer.

In light of the refined and expanded evidence, we propose a model in which stigma (and particularly its internalised form) acts as a central psychosocial stressor that increases distress and conditions adjustment and quality of life (Figure 2). This relationship is not direct, but is mediated by coping modes [41,44] and modulated by resources that act as buffers: social support [40,43,45] and self-compassion [22]. Smoking history would intervene in a twofold sense: as a factor that grades the intensity of stigma (higher in current smokers than in former smokers and in those who have never smoked) [16] and, above all, as a modulator of symptom appraisal, help-seeking, and the social attribution of blame. Nevertheless, stigma is not determined by smoking: it is also highly prevalent among those who have never smoked, which indicates that the attribution of responsibility operates on the diagnosis as a whole. Within stigma, its internalised component (guilt, shame, and self-blame) is the pathway most directly linked to depression and anxiety [38,55].

As a complementary hypothesis (not examined directly in lung cancer patients, but suggested by research in smoking populations) smoking could also be associated with prior difficulties in emotion regulation [10,11,12] that would increase vulnerability to distress; this pathway remains untested in this population and constitutes a relevant gap. The model should be interpreted with caution and is offered as a framework for future research, not as an established causal sequence.

### 4.5. Clinical Implications

The findings support the systematic assessment of stigma (and specifically of its internalised component), coping, social support, and emotional distress in all lung cancer patients, including those who have never smoked, a group in which stigma may go unnoticed, and with special attention to advanced disease [39]. On the therapeutic level, interventions focused on self-compassion [22], together with cognitive–behavioural and acceptance-based approaches aimed at reducing self-blame and promoting adaptive coping strategies, appear promising; strengthening social support (both family and peer support) constitutes a complementary target of the first order [40,43,45]. At the relational level, training professionals in non-stigmatising communication that avoids reproach and the attribution of blame [58] could reduce both perceived stigma and the delay in seeking help. Finally, the cultural variability in the sources and expression of stigma [42] underscores the need to adapt interventions to each sociocultural context.

### 4.6. Strengths and Limitations

Among the strengths, the adherence to the PRISMA 2020 statement, the selection and extraction in duplicate, the focus on lung cancer populations, and the incorporation of both quantitative evidence (including several longitudinal studies) and qualitative evidence stand out. The updating of the search has also made it possible to broaden the evidence base to Asian and Latin American contexts, which improves the transferability of the results compared with previous reviews focused on Anglo-Saxon populations. Nevertheless, several limitations must be acknowledged. First, despite the presence of longitudinal studies, much of the evidence is cross-sectional, which limits causal inference. Second, self-reported measures predominate and there is heterogeneity in the stigma instruments used (e.g., the Lung Cancer Stigma Inventory versus the Cataldo scale), which hinders direct comparison across studies. Third, this clinical and methodological heterogeneity made a meta-analysis inadvisable and required a narrative synthesis. Fourth, some studies could share a sample (the two Polish studies), so their evidence should not be considered fully independent. Fifth, although the database search was complemented by backward and forward citation searching, the principal electronic search was limited to PubMed/MEDLINE and Dialnet and did not include psychology-specific or multidisciplinary databases such as PsycINFO, CINAHL, Scopus, or Web of Science. This represents an important methodological limitation for a review focused on psychosocial constructs such as stigma and coping. Although PubMed/MEDLINE indexes many of the psycho-oncology journals in which lung cancer stigma research is published, and although citation searching was used to improve retrieval, we cannot exclude the possibility that relevant studies indexed primarily in psychological or multidisciplinary databases were missed. Sixth, restricting eligibility to studies published in English or Spanish may have introduced a language bias and may have excluded relevant evidence published in other European or Asian languages. Finally, no included study examined directly the relationship between smoking and emotion regulation in lung cancer patients, so that pathway of the model remains untested.

### 4.7. Future Research Directions

Future research should prioritise longitudinal and prospective designs that explicitly model the interaction between smoking history, stigma (perceived and internalised), coping, social support, and psychological outcomes; deliberately include patients who have never smoked; harmonise stigma measurement instruments and validate them cross-culturally; complement self-report with other sources of measurement; and evaluate, through controlled trials, interventions aimed at reducing internalised stigma and strengthening self-compassion, adaptive coping, and social support.

## 5. Conclusions and Clinical Implications

This systematic review, based on 24 studies in lung cancer populations, concludes that stigma (and in particular its internalised form) constitutes a central psychosocial factor, associated with higher levels of distress, depression, and anxiety and with worse quality of life; the available longitudinal evidence further indicates that stigma precedes and predicts distress. Contrary to what is usually assumed, stigma does not depend exclusively on smoking history and also affects those who have never smoked; smoking influences mainly symptom appraisal, help-seeking, and the social attribution of blame. Coping and social support modulate adjustment and quality of life and represent modifiable intervention targets.

From a clinical perspective, these findings support the systematic assessment of internalised stigma, coping, social support, and emotional distress in all lung cancer patients (smokers and never-smokers alike) as well as the development of interventions that combine self-compassion, the reduction in self-blame, the strengthening of social support, and non-stigmatising communication by professionals, adapted to the patient’s sociocultural context. The main gap identified is the absence of studies that analyse, in an integrated and longitudinal manner, the interaction between smoking, stigma, and coping in this population, which should be a priority direction for future research.

## Figures and Tables

**Table 1:** Characteristics of the included studies (*n* = 24).

Author (Year) [Ref.]	Country	Design	Sample	Population	Main Results	Main Conclusions
Carter-Harris et al. (2014) [56]	USA	Cross-sectional (survey + interview)	*n* = 94	Lung cancer patients	Higher perceived stigma → longer delay in seeking medical help.	Stigma may contribute to diagnostic delay.
Chambers et al. (2015) [57]	Australia	Cross-sectional	*n* = 151	Lung cancer patients	Anxiety 49%, depression 41%, distress 51%; stigma → more distress and worse QoL (mediated by social constraints and appraisals).	Stigma is a central psychosocial factor.
Shen et al. (2016) [58]	USA	Cross-sectional	*n* = 231	Lung cancer patients	Patient–provider communication is associated with perceived stigma.	Improving communication could reduce stigma.
Morrison et al. (2017) [26]	USA	Cross-sectional	*n* = 2205	Newly diagnosed patients	Emotional problems associated with worse QoL and greater symptom burden.	Psychosocial interventions may mitigate the impact.
Hamann et al. (2018) [21]	USA	Psychometric	*n* = 266	Lung cancer patients	Development and preliminary validation of the LCSI.	The LCSI validly measures stigma.
Hajdarevic et al. (2018) [59]	Sweden/UK	Qualitative	*n* = 72	Lung cancer patients	Stigma conditions how patients talk about smoking; it varies across countries.	Stigma shapes the patient’s narrative.
Williamson et al. (2018) [38]	USA	Longitudinal (12 wk)	*n* = 101	Patients on treatment	Internalised stigma and concealment → worse adjustment and QoL.	Mechanisms of worse psychological adjustment.
Chabowski et al. (2018) [36]	Poland	Cross-sectional	*n* = 185	NSCLC patients	Maladaptive coping (mini-MAC) → worse QoL.	Coping influences QoL.
Liao et al. (2018) [60]	Taiwan	Qualitative	*n* = 34	Women with incurable lung cancer	Multiple coping: social support, positive thoughts, avoidance, faith.	Coping is diverse and amenable to intervention.
Ostroff et al. (2019) [23]	USA	Cross-sectional (validation)	*n* = 266	Lung cancer patients	Stigma (LCSI) associated with depression; clinical threshold LCSI ≥ 38.	Allows identification of clinically significant stigma.
Williamson et al. (2020) [16]	USA	Cross-sectional	*n* = 266	Lung cancer patients	Stigma higher in current > former > never smokers; 60% of never-smokers with significant stigma.	Smoking grades stigma, but it is not exclusive.
Williamson et al. (2020) [55]	USA	Cross-sectional	*n* = 50	Adults with lung cancer	Guilt/shame → anxiety and depression, mediated by internalised stigma.	Reducing self-blame could ease distress.
Jankowska-Pol. et al. (2020) [29]	Poland	Cross-sectional	*n* = 185	NSCLC patients	Destructive coping → more anxiety/depression; constructive coping protective.	Cognitive adjustment modulates anxiety and depression.
van Montfort et al. (2020) [61]	Netherlands	Cross-sectional	*n* = 130	Lung cancer patients	Psychological profiles are associated with QoL.	Identifying profiles helps detect the most vulnerable.
Rose et al. (2021) [37]	Australia	Longitudinal	*n* = 194	Newly diagnosed patients	Baseline stigma predicted distress at 3 and 6 months.	Stigma precedes and predicts distress.
Williamson et al. (2022) [22]	USA	Longitudinal (12 wk)	*n* = 108	Patients on treatment	Facets of stigma predict adjustment; self-compassion moderates.	Self-compassion could attenuate the impact of stigma.
Black et al. (2022) [62]	UK	Qualitative	*n* = 40	Lung cancer patients	Symptom appraisal and help-seeking differ by smoking status.	Smoking influences pre-diagnostic behaviour.
González-Ling et al. (2023) [39]	Mexico	Cross-sectional	*n* = 204	Advanced/metastatic lung cancer patients	Relevant anxiety, depression, and distress, associated with worse QoL.	High psychological burden in advanced disease; screening needed.
Lim et al. (2024) [40]	South Korea	Cross-sectional	*n* = 184	Lung cancer patients	Stigma worsens QoL through distress; support from friends moderates.	Address stigma and distress, and reinforce social support.
Ma et al. (2024) [41]	China	Cross-sectional	*n* = 304	Lung cancer surgery patients	Medical coping modes mediate the stigma–QoL relationship.	Coping is a modifiable target between stigma and QoL.
Liu et al. (2016) [42]	China	Qualitative	*n* = 17	Lung cancer patients	Sources of stigma (tobacco, loss of working capacity, deterioration of self-image) and coping strategies such as concealing the illness.	Perceived stigma is frequent and should be addressed together with its associated factors.
Ding et al. (2024) [43]	China	Cross-sectional	*n* = 300	Lung cancer patients	Social support and coping style are associated with QoL and psychological state.	Social support and adaptive coping are targets for improvement.
Liu et al. (2025) [44]	China	Cross-sectional	*n* = 435	Lung cancer patients	Distress is associated with coping, illness perception, symptoms, and social support.	Multifactorial model useful for screening and intervention.
Xu et al. (2025) [45]	China	Cross-sectional	*n* = 390	Patients on chemotherapy	Depression reduces QoL, mediated by perceived social support.	Social support buffers the impact of depression on QoL.

NSCLC, non-small-cell lung cancer; QoL, quality of life; LCSI, Lung Cancer Stigma Inventory; mini-MAC, mini-Mental Adjustment to Cancer. Note: Chabowski et al. (2018) [36] and Jankowska-Polańska et al. (2020) [29] may derive from a single cohort (*n* = 185, same research group and instrument), so their evidence should not be regarded as fully independent.

**Table 2:** Cross-sectional observational studies (JBI Critical Appraisal Checklist for Analytical Cross-Sectional Studies).

Study [Ref.]	1	2	3	4	5	6	7	8	Overall
Carter-Harris (2014) [56]	Y	Y	Y	Y	U	U	Y	Y	Moderate
Chambers (2015) [57]	Y	Y	Y	Y	Y	U	Y	Y	Moderate-high
Shen (2016) [58]	Y	Y	Y	Y	U	Y	Y	Y	Moderate-high
Morrison (2017) [26]	Y	Y	Y	Y	Y	U	Y	Y	High
Ostroff (2019) [23]	Y	Y	Y	Y	U	U	Y	Y	Moderate-high
Williamson (2020) [16]	Y	Y	Y	Y	Y	Y	Y	Y	High
Williamson (2020) [55]	Y	Y	Y	Y	U	U	Y	Y	Moderate
Chabowski (2018) [36]	Y	Y	Y	Y	Y	U	Y	Y	Moderate-high
Jankowska-Pol. (2020) [29]	Y	Y	Y	Y	Y	Y	Y	Y	High
van Montfort (2020) [61]	Y	Y	Y	Y	U	U	Y	Y	Moderate-high
González-Ling (2023) [39]	Y	Y	Y	Y	U	U	Y	Y	Moderate-high
Lim (2024) [40]	Y	Y	Y	Y	Y	Y	Y	Y	High
Ma (2024) [41]	Y	Y	Y	Y	U	Y	Y	Y	Moderate-high
Ding (2024) [43]	Y	Y	Y	Y	U	Y	Y	Y	Moderate-high
Liu (2025) [44]	Y	Y	Y	Y	Y	Y	Y	Y	High
Xu (2025) [45]	Y	Y	Y	Y	U	Y	Y	Y	Moderate-high

JBI items (cross-sectional): 1, inclusion criteria defined; 2, subjects and setting described; 3, exposure measured in a valid and reliable way; 4, objective/standard criteria for the condition; 5, confounding factors identified; 6, strategies to deal with confounding; 7, outcomes measured in a valid and reliable way; 8, appropriate statistical analysis. Y, yes; U, unclear. Note on overall ratings: the overall judgement is not derived only from the count of “Y”/“U” responses on the eight items; it also integrates the seriousness of any “U” or limitation for the specific research question and the methodological strengths beyond the checklist (e.g., sample size, representativeness, multivariable adjustment, recruitment strategy). Main contextual limitations per study: Carter-Harris (2014) [56], small convenience sample and limited confounder adjustment (overall: moderate); Chambers (2015) [57], confounder control reported but limited multivariable modelling (moderate-high); Shen (2016) [58], confounders identified but only partial adjustment in regression models (moderate-high); Morrison (2017) [26], large multicentre sample with adequate adjustment and validated instruments (high); Ostroff (2019) [23], psychometric-validation sample with limited confounder adjustment for clinical outcomes (moderate-high); Williamson (2020) [16], well-characterised sample with explicit confounder strategy and multivariable models (high); Williamson (2020) [55], small sample with limited adjustment for clinical confounders (moderate); Chabowski (2018) [36], adequate sample with limited multivariable modelling (moderate-high); Jankowska-Pol. (2020) [29], large sample with explicit confounder strategy and multivariable models (high); van Montfort (2020) [61], adequate sample with limited multivariable modelling for the QoL outcome (moderate-high); González-Ling (2023) [39], advanced-disease sample with limited confounder adjustment (moderate-high); Lim (2024) [40], adequate sample with explicit confounder strategy (high); Ma (2024) [41], adequate sample with explicit mediation analysis but limited confounder adjustment (moderate-high); Ding (2024) [43], adequate sample with partial confounder control (moderate-high); Liu (2025) [44], large sample with multivariable modelling and explicit confounder strategy (high); Xu (2025) [45], adequate sample with mediation analysis but partial confounder adjustment (moderate-high). This rationale should be read in conjunction with the Y/U pattern in the table.

**Table 3:** Longitudinal studies (JBI Critical Appraisal Checklist for Cohort Studies).

Study [Ref.]	1	2	3	4	5	6	7	8	9	10	11	Overall	Main Limitations
Williamson (2018) [38]	Y	Y	Y	Y	U	Y	Y	Y	U	U	Y	Moderate-high	Limited sample size; brief 12-week follow-up; self-report; losses/follow-up not described in sufficient detail.
Williamson (2022) [22]	Y	Y	Y	Y	Y	Y	Y	Y	U	U	Y	Moderate-high	Brief follow-up; self-report; losses not fully detailed.
Rose (2021) [37]	Y	Y	Y	U	U	Y	Y	Y	U	U	Y	Moderate-high	Good longitudinal value, but limited by self-reports, possible loss to follow-up, and absence of an experimental design.

JBI items (cohorts): 1, groups/sample from a defined population; 2, exposure measured similarly; 3, exposure measured validly/reliably; 4, confounding factors identified; 5, strategies to deal with confounding; 6, absence of the outcome at baseline (baseline measurement); 7, outcomes measured validly/reliably; 8, follow-up time reported and sufficient; 9, complete follow-up or losses described; 10, strategies to address incomplete follow-up; 11, appropriate statistical analysis. Note: tool applied to single-cohort longitudinal designs. Y, yes; U, unclear.

**Table 4:** Qualitative studies (CASP Qualitative Studies Checklist).

Study [Ref.]	1	2	3	4	5	6	7	8	9	10	Overall	Main Limitations
Hajdarevic (2018) [59]	Y	Y	Y	Y	Y	U	Y	Y	Y	Y	High	Reflexivity (researcher–participant relationship) not made explicit.
Black (2022) [62]	Y	Y	Y	Y	Y	U	Y	Y	Y	Y	High	Reflexivity not made explicit; possible recall bias in the account of symptoms and help-seeking.
Liao (2018) [60]	Y	Y	Y	Y	Y	U	U	Y	Y	Y	Moderate-high	Reflexivity and ethical/methodological aspects less detailed; specific sample of women with incurable disease.
Liu et al. (2016) [42]	Y	Y	Y	Y	Y	U	Y	Y	Y	Y	High	Reflexivity underdeveloped; small, purposive sample from a single Chinese hospital; data collected in hospitalised patients.

CASP items (qualitative): 1, clear aims; 2, qualitative methodology appropriate; 3, design appropriate to the aims; 4, appropriate sampling strategy; 5, appropriate data collection; 6, reflexivity (researcher–participant relationship); 7, consideration of ethical issues; 8, sufficiently rigorous analysis; 9, clear statement of findings; 10, value/contribution of the research. Y, yes; U, unclear.

## Data Availability

No new data were created or analyzed in this study.

## Supplementary Materials

The following supporting information can be downloaded at: https://www.mdpi.com/article/10.3390/curroncol33070408/s1, File S1: Search strategies (complete search equations for each database, reported in accordance with the PRISMA-S extension; File S2: completed PRISMA 2020 checklist.

## Abbreviations

The following abbreviations are used in this manuscript:

AECC	Spanish Association Against Cancer (Asociación Española Contra el Cáncer)
CASP	Critical Appraisal Skills Programme
COSMIN	Consensus-based Standards for the selection of health Measurement Instruments
GLOBOCAN	Global Cancer Observatory (global cancer incidence and mortality estimates)
HADS	Hospital Anxiety and Depression Scale
IARC	International Agency for Research on Cancer
JBI	Joanna Briggs Institute
LCSI	Lung Cancer Stigma Inventory
MeSH	Medical Subject Headings
mini-MAC	mini-Mental Adjustment to Cancer (scale)
MMAT	Mixed Methods Appraisal Tool
NCCN	National Comprehensive Cancer Network
NSCLC	non-small-cell lung cancer
OSF	Open Science Framework
PICO	Population, Intervention, Comparison, Outcome
PRISMA	Preferred Reporting Items for Systematic Reviews and Meta-Analyses
PRISMA-S	PRISMA extension for Searching
QoL	quality of life
SCLC	small-cell lung cancer
SF-8	Short Form-8 Health Survey
SPIDER	Sample, Phenomenon of Interest, Design, Evaluation, Research type
SWiM	Synthesis Without Meta-analysis
WHO	World Health Organization
