# Supplementary material for: Tobacco Use, Stigma, and Coping in Lung Cancer: A Systematic Review of Their Psychosocial Interactions and Clinical Implications
Source: Curr Oncol. 2026 Jul 9;33(7):408. doi: 10.3390/curroncol33070408 (PMC13409720; doi:10.3390/curroncol33070408)
Supplement: Supplementary file 1 [file curroncol-33-00408-s001.zip › S1 Search strategies.pdf]

Supplementary Material

S1. Search strategies

Reported in accordance with the PRISMA-S extension [41]

Searches were carried out in PubMed/MEDLINE and Dialnet. For each database, the complete strategy, the date of execution, and the number of records retrieved are reported. The temporal (1 January 2014 - 30 April 2026) and language (English and Spanish) limits described in Section 2.3 were applied. The mandatory combination was “lung cancer” AND (stigma OR coping OR psychological outcomes); the tobacco block was used as an optional term for the third axis and not as a restrictive filter.

1. PubMed / MEDLINE

Records retrieved: n = 6747

Set A. Lung cancer (population)

```
A1.  "Lung Neoplasms"[Mesh]
A2.  lung[tiab] AND (cancer*[tiab] OR carcinoma*[tiab] OR neoplas*[tiab] OR tumo*[tiab] OR adenocarcinoma*[tiab])
A3.  NSCLC[tiab] OR SCLC[tiab] OR "non-small cell lung"[tiab] OR "small cell lung"[tiab]
A4.  A1 OR A2 OR A3
```

Set B. Stigma

```
B1.  "Social Stigma"[Mesh] OR "Stereotyping"[Mesh]
B2.  stigma*[tiab] OR stigmati*[tiab] OR shame[tiab] OR guilt[tiab] OR blame[tiab] OR "self-blame"[tiab]
B3.  B1 OR B2
```

Set C. Coping

```
C1.  "Adaptation, Psychological"[Mesh]
C2.  coping[tiab] OR "coping strateg*"[tiab] OR "coping style*"[tiab] OR "psychological adjustment"[tiab] OR "emotion* regulation"[tiab]
C3.  C1 OR C2
```

Set D. Psychological outcomes

```
D1.  "Stress, Psychological"[Mesh] OR "Psychological Distress"[Mesh] OR "Depression"[Mesh] OR "Depressive Disorder"[Mesh] OR "Anxiety"[Mesh] OR "Quality of Life"[Mesh]
D2.  distress[tiab] OR depress*[tiab] OR anxiety[tiab] OR anxious[tiab] OR "quality of life"[tiab] OR "psychological well-being"[tiab] OR "psychological wellbeing"[tiab]
D3.  D1 OR D2
```

Set E. Smoking (optional; axis 3)

```
E1.  "Smoking"[Mesh] OR "Tobacco Use"[Mesh] OR "Tobacco Smoking"[Mesh] OR "Smokers"[Mesh]
E2.  smoking[tiab] OR tobacco[tiab] OR smoker*[tiab] OR nicotine[tiab]
E3.  E1 OR E2
```

Final combination

```
F1.  A4 AND (B3 OR C3 OR D3)
```

Filters applied: ("2014/01/01"[Date - Publication]: "2026/04/30"[Date - Publication]) AND Humans[Filter] AND (English[lang] OR Spanish[lang])

Full equation

```
("Lung Neoplasms"[Mesh] OR (lung[tiab] AND (cancer*[tiab] OR carcinoma*[tiab] OR
neoplas*[tiab] OR tumo*[tiab] OR adenocarcinoma*[tiab])) OR NSCLC[tiab] OR SCLC[tiab] OR
"non-small cell lung"[tiab] OR "small cell lung"[tiab]) AND ("Social Stigma"[Mesh] OR
"Stereotyping"[Mesh] OR stigma*[tiab] OR stigmati*[tiab] OR shame[tiab] OR guilt[tiab] OR
blame[tiab] OR "self-blame"[tiab] OR "Adaptation, Psychological"[Mesh] OR coping[tiab] OR
```

```
"coping strateg*"[tiab] OR "coping style*"[tiab] OR "psychological adjustment"[tiab] OR
"emotion* regulation"[tiab] OR "Stress, Psychological"[Mesh] OR "Psychological
Distress"[Mesh] OR "Depression"[Mesh] OR "Depressive Disorder"[Mesh] OR "Anxiety"[Mesh] OR
"Quality of Life"[Mesh] OR distress[tiab] OR depress*[tiab] OR anxiety[tiab] OR anxious[tiab]
OR "quality of life"[tiab] OR "psychological well-being"[tiab] OR "psychological
wellbeing"[tiab]) AND ("2014/01/01"[Date - Publication] : "2026/04/30"[Date - Publication])
AND (English[lang] OR Spanish[lang]) AND Humans[Filter]
```

2. Dialnet

Records retrieved: n = 655

Dialnet offers limited Boolean operators and does not support controlled vocabulary (MeSH); therefore, the search was run using combinations of terms in the title and abstract fields, with a 2014-2026 date filter and Spanish language. The combinations used are documented below (with and without accents, owing to indexing variants). The search terms are kept in Spanish, as this is the language of the database in which the search was executed.

Search combinations

```
D-1.  "cáncer de pulmón" AND estigma
D-2.  "cáncer de pulmón" AND afrontamiento
D-3.  "cáncer de pulmón" AND ("calidad de vida" OR distrés OR ansiedad OR depresión)
D-4.  ("cáncer de pulmón" OR "carcinoma broncogénico" OR "neoplasia pulmonar") AND
(tabaquismo OR tabaco)
D-5.  "cancer de pulmon" AND (estigma OR afrontamiento)   [variant without accents]
```
